# Global and national public awareness and interest in glomerular diseases from 2004 to 2024

**DOI:** 10.3389/fneph.2025.1519481

**Published:** 2025-01-23

**Authors:** Suryanarayanan Balakrishnan, Charat Thongprayoon, Iasmina M. Craici, Wisit Cheungpasitporn, Jing Miao

**Affiliations:** Division of Nephrology and Hypertension, Mayo Clinic, Rochester, MN, United States

**Keywords:** IgA nephropathy, membranous glomerulonephritis, focal segmental glomerulosclerosis, lupus nephritis, diabetic nephropathy, public awareness

## Abstract

**Background:**

Glomerular diseases significantly impact global health. This study investigated public interest in five common glomerular diseases.

**Methods:**

Google Trends™ were used to analyze search activity from January 2004 to December 2024 for IgA nephropathy (IgAN), membranous glomerulonephritis (MN), focal segmental glomerulosclerosis (FSGS), lupus nephritis (LN), and diabetic nephropathy (DN). Data were retrieved both globally and in English-speaking countries, including the United States. Monthly and yearly relative search activity were assessed and compared.

**Results:**

Globally, IgAN had the highest average relative search activity, followed by DN, FSGS, LN, and MN. Both IgAN and FSGS exhibited declining trends, while LN showed an upward pattern. MN and DN experienced a modest decline before 2016, preceded by a slight increase. Among English-speaking countries, search interest was predominantly concentrated in five countries, primarily including the United States, United Kingdom, Canada, and Australia, with the United States consistently ranking as the leading country. For IgAN, LN, and MN, the trends observed in the United States appeared to align with global data. In contrast, search interest for FSGS exceeded global levels, while interest in DN was slightly lower than global activity. In the United States, IgAN, FSGS, and LN were most prominent in North Dakota, Massachusetts, and Delaware, respectively, while DN and MN saw peak activity in West Virginia.

**Conclusion:**

Public engagement with glomerular diseases has not uniformly grown, at least in English-speaking countries, emphasizing the need for enhanced awareness efforts. Future analysis should prioritize search terms in the predominant language of each country.

## 1 Introduction

Glomerular diseases affect millions worldwide and impose substantial burdens on healthcare systems (1). These conditions can cause significant kidney function decline, leading to chronic kidney disease (CKD) and end-stage kidney disease (ESKD). The most common types of glomerulonephritis vary based on geographical region and age group (2, 3), but several forms are frequently diagnosed worldwide, including IgA nephropathy (IgAN), membranous glomerulonephritis (MN), focal segmental glomerulosclerosis (FSGS). In the United States, glomerulonephritis is estimated to account for about 10 - 15% of all ESKD thereby making it the third leading cause of ESKD (4, 5) with a significant financial burden associated with many of these diseases such as lupus nephritis (LN) (6). The incidence of diabetic nephropathy (DN) is increasing; added to this is the fact the global burden of CKD due to glomerulonephritis has increased by 77% since 1990 with much of this rise occurring in places with low sociodemographic index (7).

Understanding and raising awareness of these diseases is vital, particularly as new treatments and clinical trials offer promising avenues for improved patient care. Raising public awareness and engagement regarding glomerular diseases can promote earlier diagnosis and treatment, enhancing patient outcomes and reducing healthcare burdens. Community engagement can also inspire individuals to participate in clinical trials, which are crucial for advancing new treatments and improving patient care. Public involvement fosters educational initiatives, equipping patients with knowledge about their conditions and treatment options. Recognizing trends in community interest enables healthcare providers and policymakers to allocate resources more efficiently and assists researchers and pharmaceutical companies in designing targeted therapies and conducting relevant clinical trials. Google Trends, the most often used tool, can track changes in search behavior in various conditions, such as COVID (8), musculoskeletal pain (9), benign prostatic hyperplasia (10), urological cancer (11), renal replacement therapy (12, 13), and kidney stones (14).

Glomerular diseases have a profound impact, yet public awareness and interest in these conditions remain poorly understood. This study aims to fill this gap by exploring public engagement in five common glomerular diseases through the analysis of Google Trends data. By examining these patterns, we can pinpoint areas of limited awareness and design focused interventions to enhance education and understanding of these diseases.

## 2 Methods

### 2.1 Retrieving Google trends data on glomerular diseases

Google Trends, a free online tool (15), offers monthly data on internet search queries dating back to 2004. This makes it an invaluable resource for tracking patterns and trends in Google search activity, enabling an investigation and understanding of public interest over time. In Google Trends, the numbers indicate search activity relative to the highest point on the chart for the given region and period. A value of 100 represents peak popularity, while 50 indicates the term is half as popular, and 0 means insufficient data for that term.

In this study, Google Trends, accessed on December 24, 2024, was used to analyze public interest, both globally and in the regions where English is the official or primary language, including the United States, in five common glomerular diseases from January 2004 to May 13, 2024 (all data updated in December 24, 2024). The terms used were “IgA nephropathy (Disease),” “Membranous glomerulonephritis (Disease),” “Focal segmental glomerulosclerosis (Syndrome),” “Lupus nephritis (Disease),” and “Diabetic nephropathy (Disease)” (**Supplementary Figure S1**). Searches were conducted in English, the primary language of medical research.

This study is in accordance with Google’s privacy policy (16). The Google Trends data used cannot be traced back to individual users. The database does not retain any personal information, such as the identity, internet protocol address, or specific location of users. Additionally, Google anonymizes any original web search logs older than nine months, further ensuring the privacy and confidentiality of online search behaviors.

### 2.2 Data analysis

The relative search interest (RSI) data for each glomerular disease was obtained from Google Trends on a monthly basis. Annual average RSI values were calculated to assess year-to-year changes in search activity. Monthly RSI trends were also analyzed and presented in the **Supplementary Materials**. We conducted subregional analysis of search interest globally in countries where English is the official or primary language, as well as within the United States at the state level.

The Augmented Dickey–Fuller (ADF) test was used to check the stationarity of a time series; the significance of the test is 0.05. The p value less than 0.05 implies that the relative search interest does not exhibit seasonality that would affect its mean or variance over time, indicating that the public interest in these terms has been consistent, without any long-term upward or downward drift. The comparative analysis of search trends across these diseases was performed using the ANOVA test on the slopes/trends extracted by fit linear regression model to determine if there are statistically significant differences between the trends of the time series. The average search interests by both yearly and monthly across these diseases were compared using one-way ANOVA with two-sided multiple comparisons. Additionally, a trends analysis for the upcoming 12 months was conducted using Time Series Forecasting. The JMP Pro 18.1.1 (SAS Institute, Inc.) and Prism 8.2.1 (GraphPad Software, Inc.) were used for statistical analysis and graph creation.

## 3 Results

### 3.1 Global search interest in glomerular diseases

**Figure 1:**
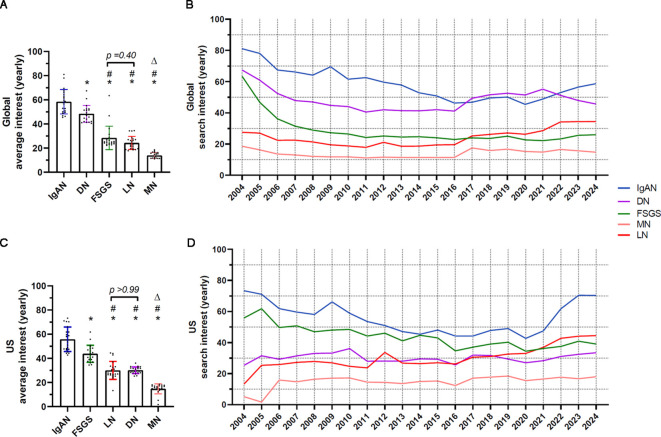
Yearly data on search trends for five glomerular diseases. Yearly data on global and United States search activity for five glomerular diseases. **(A)** Average yearly global search activity. ^*^p<0.0001 vs IgAN, ^#^p<0.0001 vs DN, ^Δ^p<0.0001 vs FSGS and LN. **(B)** Yearly data on global trends. **(C)** Average yearly U.S search activity. ^*^p<0.0001 vs IgAN, ^#^p<0.0001 vs FSGS, ^Δ^p<0.0001 vs DN and LN. **(D)** Yearly data on United States search trends. IgAN, IgA nephropathy; MN, membranous glomerulonephritis; FSGS, focal segmental glomerulosclerosis; LN, lupus nephritis; DN, diabetic nephropathy.

Globally, the mean yearly RSI scores over the 21-year period varied significantly (**Figure 1A**; **Table 1**), with IgAN leading (58.4 ± 10.1), followed by DN (48.4 ± 7.0), FSGS (28.5 ± 9.8), and LN (24.3 ± 5.4). MN had the lowest score (13.9 ± 2.4). As shown in **Figure 1B** and **Supplementary Figure S2**, IgAN, DN, and FSGS initially had significantly higher search volumes compared to LN and MN. IgAN showed fluctuating search activity, with a noticeable drop from 2004 to 2016, followed by modest growth with intermittent peaks and dips from 2017 onward. DN exhibited an overall downward trend from 2004 to 2016, followed by a marked recovery until 2021, after which it experienced another downturn. FSGS begun with relatively high search activity but saw a sharp decline after 2004, eventually stabilizing at a lower level. MN consistently had the lowest search volume ranging between 15 and 20 among the diseases, remaining relatively stable with a small jump since 2016. LN demonstrated a gradual downward trend from 2004 to 2016, followed by a moderate growth from 2016 to 2020, and a substantial rise from 2021 onward.

**Table 1:** Global search interest for five glomerular diseases.

	IgAN	DN	LN	FSGS	MN
Average interest (yearly)	58.4 ± 10.1	48.4 ± 7.0	24.3 ± 5.4	28.5 ± 9.8	13.9 ± 2.4
Peak interest and date (year)	81.1 (2004)	67.0 (2004)	34.4 (2024)	63.0 (2004)	18.6 (2004)
Lowest interest and date (year)	45.4 (2020)	40.5 (2011)	17.8 (2011)	22.2 (2021)	11.1 (2011)
Most recent interest (2024)	58.7	45.7	34.4	25.9	14.8
Interest by region – the English-speaking countries with the average monthly RSI > 1	US (55.79)	US (30.15)	US (30.00)	US (43.76)	US (14.79)
	UK (31.03)	UK (14.87)	UK (7.50)	UK (11.43)	UK (5.10)
	Canada (18.72)	India (8.88)	Canada (4.45)	Canada (8.05)	Canada (1.85)
	Australia (10.04)	Canada (7.02)	India (3.32)	Australia (4.14)	India (1.58)
	Ireland (6.58)	Australia (5.65)	Philippines (2.90)	India (3.53)	Australia (1.40)
	India (4.55)	Philippines (3.75)	Australia (2.00)		
	Philippines (3.85)	New Zealand (1.80)	South Africa (1.51)		
	New Zealand (3.79)	Pakistan (1.61)			
	Singapore (3.19)	Singapore (1.51)			

The comparative analysis of global yearly data reveals significant variations (p<0.0001) in search trends for glomerular diseases over time. IgAN and FSGS exhibited a marked decline, while LN demonstrated a notable upward trend. MN and DN experienced a modest decline before 2016, preceded by a slight increase accompanied by minor variations.

### 3.2 Search activity in English-speaking countries

Since the analysis utilized English terms, the extracted data may not fully represent global trends. To address this, search activity data were specifically collected from countries where English is the official or primary language. For each glomerular disease, the average monthly RSI was calculated for these countries. The results identified 11 countries with an average monthly RSI exceeding 1, and with the United States consistently ranking the leading country exhibiting the highest search activity (**Table 1**).

In the United States, the mean yearly RSI scores over the past 21 years varied significantly (**Figure 1C**; **Table 2**), with IgAN ranking highest (55.8 ± 10.2), followed by FSGS (43.8 ± 7.1), DN (30.2 ± 2.8), and LN (30.0 ± 7.4). MN had the lowest score (14.8 ± 4.1). As shown in **Figure 1D** and **Supplementary Figure S3**, search trends for the five glomerular diseases exhibited distinct patterns compared to global data. IgAN and FSGS showed higher levels of search interest compared to the other three diseases. Specifically, IgAN experienced a consistent decline until 2020, followed by a sharp rebound, reaching its earlier levels by 2023. FSGS initially displayed a significant rise but subsequently entered a stable downward phase with occasional fluctuations. DN, MN, and LN begun with lower levels of search activity. DN and MN demonstrated a relatively stable trend, but LN showed a minor upward pattern. Similarly, the analysis of yearly data from the United States highlights statistically significant (p<0.0001) differences in trends among the glomerular diseases. IgAN, MN, and DN displayed upward trajectories, whereas FSGS showed a declining pattern. LN remained relatively stable, with only minor changes in activity over the years.

**Table 2:** Search interest for five glomerular diseases in the United States.

	IgAN	DN	LN	FSGS	MN
Average interest (yearly)	55.8 ± 10.2	30.2 ± 2.8	30.0 ± 7.4	43.8 ± 7.1	14.8 ± 4.1
Peak interest and date (year)	73.3 (2004)	36.1 (2010)	44.4 (2024)	61.8 (2005)	18.4 (2019)
Lowest interest and date (year)	42.7 (2020)	25.33 (2004)	13.3 (2004)	34.4 (2020)	1.7 (2005)
Most recent interest (2024) ^a^	70.3	33.4	44.4	39.0	18.0
Interest by region -top 5 states ^b^	North Dakota (100)	West Virginia (100)	Delaware (100)	Massachusetts (100)	West Virginia (100)
	Massachusetts (94)	Massachusetts (100)	Connecticut (100)	Pennsylvania (100)	Connecticut (83)
	Michigan (94)	Connecticut (88)	Mississippi (100)	Minnesota (100)	Massachusetts (83)
	West Virginia (94)	Mississippi (88)	Massachusetts (90)	Maryland (91)	Vermont (83)
	South Dakota 94)	Kentucky (88)	New York (90)	Missouri (91)	Michigan (66)

a Most recent interest indicates the year 2024 (calculated from Jan to December 24).

b See in which location the term was most popular during the specified time frame. Values are calculated on a scale from 0 to 100, where 100 is the location with the most popularity as a fraction of total searches in that location, a value of 50 indicates a location which is half as popular. A value of 0 indicates a location where there was not enough data for this term. Note: A higher value means a higher proportion of all queries, not a higher absolute query count. So, a tiny country where 80% of the queries are for “bananas” will get twice the score of a giant country where only 40% of the queries are for “bananas”.

The top five English-speaking countries, where the search term demonstrated the highest popularity (most RSI values exceeding 5), were selected for further analysis. In the case of IgAN (**Supplementary Figure S4**), the highest search activity was observed in the United States, United Kingdom, Canada, Australia, and Ireland. In the UNITED STATES, search activity closely mirrored global trends. The Pearson correlation coefficient between the Global and United States data is approximately 0.73, indicating a moderate positive correlation and suggesting similar trends across these datasets. Notable variations in search activity were evident before 2011 in the United Kingdom, Canada, and Australia. Although search interest in Ireland was generally lower, it experienced significant fluctuations during the specified period.

For FSGS (**Supplementary Figure S5**), the top 5 countries with the highest search popularity were the United States, United Kingdom, Canada, Australia, and India. The bulk of the search activity centered in the United States, where the RSI was higher than the global average. The Pearson correlation coefficient between the Global and United States data is approximately 0.65, indicating a moderate positive correlation and pointing to similar patterns between these datasets. Search activity in the United Kingdom, Canada, Australia, and India were significantly lower, with notable fluctuations occurring before 2012. After this period, the RSI in these countries remained below 20.

For DN (**Supplementary Figure S6**), the United States, United Kingdom, India, Canada, and Australia were identified as the top five English-speaking countries with the highest frequency of searches for the term. All search activity was lower than the global trends. The Pearson correlation coefficient between the Global and United States data is approximately 0.40, suggesting a weak positive correlation within these datasets.

For LN (**Supplementary Figure S7**), the United States, United Kingdom, Canada, India, and Philippines were identified as the top five English-speaking countries with the highest frequency of searches for the term. Search activity was generally low across these countries. In the United States, search trends closely aligned with global patterns. The Pearson correlation coefficient between the Global and United States data is approximately 0.62, suggesting a moderate positive correlation and pointing to similar trends within these datasets. In other four countries, the RSI was below 20.

In the case of MN (**Supplementary Figure S8**), the United States, United Kingdom, Canada, India, and Australia were identified as the top five English-speaking countries with the highest frequency of searches for the term. Search activity was generally low across these countries, with the RSI remaining below 30. In the United States, search trends closely aligned with global patterns post-2007. The Pearson correlation coefficient between the Global and United States data is approximately 0.58, suggesting a moderate positive correlation and pointing to similar trends within these datasets.

### 3.3 Time series forecasting analysis

Time series forecasting analysis indicates that no significant increases are expected in the predicted trends for the next 12 months within 2025 across the five glomerular diseases studied, both globally and in the United States (**Supplementary Figures S2**, **S3**).

### 3.4 Differentiation of geographic interest search in the United States

In the United States, search activities for the five glomerular diseases varied significantly across states (**Supplementary Figure S9**; **Table 2**). North Dakota recorded the most searches for IgAN. West Virginia exhibited strongest engagement for DN and MN. Massachusetts led in FSGS-related searches and, was among the top five states for IgAN, DN and LN.

### 3.5 ADF stationarity analysis of yearly search activity

The ADF test results (**Table 3**) suggest that global search trends over the time series for FSGS, LN and DN are stationary (ADF statistic = -4.130, -6.469, and -3.464, p=0.001, 0.000, and 0.01, respectively), and MN potentially stationary at the 10% significance level (ADF statistic = -2.406, p=0.140). In contrast, IgAN exhibit non-stationary trends (ADF statistic = -1.171, p=0.686), indicating fluctuating patterns in public engagement with these terms.

**Table 3:** The Augmented Dickey–Fuller stationarity test of search interest trends on the yearly data for five glomerular diseases.

	IgAN	MN	FSGS	LN	DN
Global					
ADF statistic	-1.171	-2.406	-4.130	-6.469	-3.464
P value	0.686	0.140 **^a^**	**0.001 ^b^**	**0.000 ^b^**	**0.01 ^b^**
United States					
ADF statistic	-1.201	-3.135	-2.407	0.242	-3.508
P value	0.673	**0.024 ^b^**	0.139 **^a^**	0.975	**0.008 ^b^**

ADF, Augmented Dickey–Fuller.

**^a^** The search trends over the time series are potentially stationary at the 10% level.

**^b^** The p value less than 0.05 indicates that the search trends over **t**he time series are stationary.

In the United States, the trends for MN and DN are stationary (ADF statistic = -3.135 and -3.508, p=0.024 and 0.008, respectively), and FSGS potentially stationary at the 10% significance level (ADF statistic = -2.407, p=0.139); whereas IgAN and LN display non-stationary behavior (ADF statistic = -1.201 and 0.242, p=0.673 and 0.975, respectively), reflecting inconsistencies in search patterns over time.

## 4 Discussion

In the modern era, the internet has become the dominant source for accessing information, with platforms like Google serving a pivotal role. This widespread reliance on digital resources can often be seen as a reflection of increasing public engagement and curiosity. As individuals seek out more information online, it contributes to enhanced awareness across a wide range of topics. This digital flow of information not only depends individual knowledge but also fosters a more informed society, capable of engaging in more meaningful discussions and informed decision-making.

The search trends for glomerular diseases explored from 2004 to 2024 exhibit distinct patterns, reflecting not only varying levels of public awareness, but also highlighting a discrepancy between search activities and their actual incidence and prevalence. Before beginning the discussion, we want to emphasize that the search terms used in our analysis were in English. As a result, the extracted data may not fully represent global trends. Our findings from English-speaking countries indicate that search interest was predominantly concentrated in the five countries, primarily including the United States, United Kingdom, Canada, and Australia, with the United States consistently ranking as the leading country in search activity. For IgAN, LN, and MN, the trends observed in the United States appeared to align closely with global data. In contrast, search interest for FSGS in the United States exceeded global levels, while interest in DN was slightly lower than global activity. It is important to note that the global data or trends presented in this study should be viewed as a reference or illustrative example. Future research should prioritize adapting search terms to the predominant language of each country to more accurately capture public awareness and interest in specific diseases within individual regions. This approach would provide a more nuanced and accurate understanding of disease-related search behavior globally.

Both global and United States search interest in IgAN has declined over the period, while search activity in other English-speaking countries, such as the United Kingdom, Canada, and Australia, has remained relatively stable. These trends contrast with IgAN’s relatively high incidence and prevalence, as it is the most prevalent form of primary glomerulonephritis worldwide. In Europe, the annual incidence of IgAN is approximately 7.6 per million, with a point prevalence of 25.3 per million (17). Conversely, the incidence in the United States is substantially higher, estimated at 21-22 per million in 2021, with a pooled prevalence ranging between 542 and 627 per million (18). In the United States, search activity for IgAN initially fell but saw a significant resurgence in 2020, gradually returning to prior levels by 2023. Despite recent initiatives to enhance visibility and public health education through social media campaigns and the efforts of patient advocacy organizations such as the IgA Nephropathy Foundation (19), public awareness remains insufficient. In addition, IgAN is more prevalent in Asian population, as evidenced by Japan, where the incidence rate is reported at 45 cases per million population annually (20). This high rate correlates with the peak search activity for IgAN observed in Japan.

DN, the leading cause of CKD and ESKD globally, significantly impacts the diabetic population as a major diabetes complication. The prevalence of DN shows global variation; for instance, a study in Saudi Arabia found a prevalence of 10.8% among type 2 diabetes patients, with differences reflecting age and diabetes duration (21). In contrast, a systematic review and meta-analysis in North America reported a pooled prevalence of 28.2% among diabetic patients, with discrepancies observed across various countries (22). Despite these numbers, search interest in DN has mostly shown a slight decline, although there was a minor uptick between 2016 and 2021. In the United States, the prevalence of DN among adults with diabetes has remained relatively unchanged over the years. An analysis of data from the National Health and Nutrition Examination Surveys (NHANES) from 1988 to 2014 indicated that the prevalence of DN was about 26.2% in the 2009-2014 period, virtually unchanged from 28.4% in 1988-1994 (23). Global data indicates that search activity for DN experienced a slight increase after 2016, following an initial minor decline. In English-speaking countries, including the United States and United Kingdom—where the search term demonstrated the highest popularity—interest in DN remained lower than global levels and has stayed relatively stable over time. These findings highlight the need to enhance patient awareness to bridge the gap between the number of DN patients and public engagement. The American Diabetes Association (ADA) and Kidney Disease: Improving Global Outcomes (KDIGO) emphasize the critical need of screening and managing CKD in diabetic patients to slow its progression and reduce related complications (24). But simultaneously, boosting visibility through social media campaigns, support from patient advocacy groups, and increased educational efforts by healthcare providers is crucial in elevating awareness, enhancing community support, and advancing research for improved and innovative treatments (25).

LN is a common type of secondary glomerulonephritis, especially among women and certain ethnic groups. The American College of Rheumatology (ACR) guidelines report that about 35% of adults with systemic lupus erythematosus (SLE) present with clinical signs of nephritis at diagnosis, and approximately 50-60% develop nephritis within the first decade of the disease (26). A population-based study in the United States recorded an average annual incidence of 10 per million population between 1976 and 2018, with the incidence rising from 7 to 13 per million over this period (27). Similarly, the prevalence of LN rose from 168 per million in 1985 to 212 per million in 2015 (27). Correspondingly, interest in LN, as tracked by Google Trends, has increased both globally and in the United States, particularly after 2021, despite the overall search levels remained low, with the RSI less than 50. This trend suggests that recent public health efforts and media exposure have effectively heightened awareness of this condition. In 2021, the American Kidney Found, in collaboration with GSK, launched a campaign to educate the public about LN (28). The initiative focuses on improving recognition of its signs, symptoms, and treatments, aiming to facilitate early detection and better disease management. Research highlights the importance of culturally tailored education in raising awareness, addressing stigma, and combating misinformation, particularly among high-risk groups like Afro-Americans (29). The CDC and Lupus Foundation of America’s National Public Health Agenda for Lupus prioritizes strategies to enhance understanding of lupus, stressing early diagnosis and treatment (30). To further these efforts, the Lupus Foundation of America has revamped its awareness campaign, featuring real-life stories of women with lupus to empower at-risk groups, especially Afro-Americans and Hispanic/Latina women. Public health authorities should continue to leverage these approaches to maintain and expand awareness levels, ensuring early diagnosis and effective disease management remain a priority (31).

FSGS are common kidney diseases that can lead to ESKD if left untreated. The incidence and prevalence of FSGS vary across populations and regions. While there has been an upward trend in the prevalence of FSGS in Western countries, the trends are inconsistent t in Europe and Asia, and there is a decreasing trend in Far Eastern countries like China (32). In the United States, the incidence of FSGS is approximately 7 per million population annually (33). Among United States veterans, the average annual incidence and prevalence during 2000-2020 were 19.6 and 164.7 per million veterans, respectively (34). Our data shows that both global and United States search interest in FSGS has experienced a slight decline over time. In other English-speaking countries, such as the United Kingdom, Canada, and Australia, engagement levels remained very low, though their trends have been relatively stable. Although new treatments and clinical trials offer hope, the insufficient public awareness and engagement could impede efforts to improve patient outcomes (35). Additionally, there is a notable deficiency in support systems, educational materials, and patient advocacy groups specifically targeting FSGS. Elevating public health priorities in these areas could foster the development and distribution of such resources, thereby improving management strategies and the quality of life for patients with FSGS and their families.

While the overall prevalence of MN is low, it remains the predominant cause of nephrotic syndrome in adults in certain regions. The incidence of primary MN is stable but less frequent compared to FSGS or IgAN. The incidence of secondary MN, which may be associated with malignancies, infections, or autoimmune conditions, differs geographically. A systematic review reported the global incidence of MN is 10-12 per million per year among adults (36). Specifically, in Olmsted County, Minnesota, USA, the annual incidence rate of MN was documented at 10 per million between 1994 and 2003 (37). Despite these statistics, public awareness and interest in MN are relatively low both globally and in English-speaking countries such as the United States, United Kingdom, Canada, India, and Australia where the search term demonstrated the highest popularity. In the United Kingdom, the prevalence has been reported at 199 cases per million (38). The United Kingdom Kidney Association’s MN Rare Disease Group in United Kingdom plays a crucial role in raising awareness and supporting MN patients (39). This organization empower patients by offering information and resources on the disease, including symptoms and treatment options. Personal stories and testimonials shared on the United Kingdom Kidney Association’s website personalize the condition and highlight the daily challenges of those affected by MN. Furthermore, international collaborations initiated by the MN Rare Disease Group are vital for advancing research and the development of new treatments (39–41). Such efforts also potentially increase patient access to innovative therapies.

We acknowledge the limitations of this study. First, the English terms in Google Trends queries used did not reflect search behavior in non-English speaking populations. While English is standard in the medical field, general population searches often use native languages, leading to potential bias in non-English speaking regions. This limits the value of aggregated data for cross-country comparisons and trends outside English-speaking areas. Again, future research should incorporate multilingual search terms to capture linguistic diversity or focus on the predominant language of each country or region. This approach would help minimize bias and enhance the accuracy of interpreting population interest trends within specific regions. Second, the most prevalent terms associated with each disease were used to analyze public interest via Google Trends. However, we recognize that the choice of terminology can significantly influence the outcomes due to the use of synonyms, acronyms, and the naming of disease subtypes. The influence on results can also vary significantly when using different categorizations in Google Trends, even if the terminology remains the same. For instance, “focal segmental glomerulosclerosis” not only appears under various categorizations like Syndrome, Search Terms, and Topics, but also as Disease. It includes well-established acronyms like FSGS and encompasses subtypes such as APOL1-associated and HIV-associated nephropathy. This highlights the complexity and importance of carefully selecting search terms and their categorizations in the medical field. To thoroughly assess search activity in specific diseases, further detailed studies should incorporate a broad spectrum of terminologies and account for all disease subtypes. Moreover, this study does not consider regional variations or differences in information-seeking behaviors, particularly among young adults heavily influenced by social media platforms (42, 43). These variations may substantially impact the generalizability of the findings. Additionally, we were unable to thoroughly assess specific factors or analyze predefined subgroups that could affect the robustness of the data, such as age, gender, socioeconomic status, and potential barriers to internet access. Furthermore, while search trends can reflect public awareness or engagement, they do not necessarily mirror the actual epidemiological data of these conditions. The potential correlation between Google Trends data and the incidence and prevalence of glomerular diseases over time has not been unexplored due to the lack of original and reliable longitudinal data for comparison. While some studies demonstrate a correlation statistic greater than 0.7 when comparing Google Trends data to real-world epidemiology, discrepancies remain that necessitate cautious interpretation of these findings. This suggests that relying solely on Google Trends for assessing public interest presents certain biases and limitations. Notably, the digital landscape of health information-seeking behavior can be influenced by external factors, including modifications to search engine algorithms. Therefore, it is crucial to enhance Google Trends data with additional sources such as other platforms or channels, surveys, interviews, or clinical data to gain a comprehensive understanding of public interest in glomerular diseases. In addition, search activity alone does not provide a comprehensive measure of public engagement or the quality of the information accessed. Evaluating the knowledge acquisition of the general population regarding the causes, symptoms, and treatments of glomerular disease would be a valuable direction for future research. Such studies could inform the development of more effective educational media, tools, and communication strategies to improve patient understanding and engagement.

In summary, despite advancements in treatments and an increase in clinical trials for glomerular diseases, our analysis of Google Trends data indicates that public interest, especially in conditions like MN and FSGS, has not significantly grown, at least in English-speaking countries. Given the inherent limitations and potential biases of Google Trends, its reliability should be validated against reference data sources. Moreover, future analysis should prioritize search terms in the predominant language of each country. Google Trends data should be viewed as a supplemental tool and integrated with other information sources to enable more accurate and comprehensive conclusions.

## Data Availability

The original contributions presented in the study are included in the article/**Supplementary Material**. Further inquiries can be directed to the corresponding author.
