# Supplementary material for: Global and national public awareness and interest in glomerular diseases from 2004 to 2024
Source: Front Nephrol. 2025 Jan 23;5:1519481. doi: 10.3389/fneph.2025.1519481 (PMC11799545; doi:10.3389/fneph.2025.1519481)
Supplement: Supplementary file 1 [file DataSheet1.pdf]

Supplementary Material

1 Supplementary Table

Table S1. Brief overview of global and US incidence and prevalence of glomerular diseases.

	Global		US	
	**Incidence (per million per year)**	**Prevalence**	**Incidence**	**Prevalence**
DN		28.2% in DM popularity (North Americans): [1]<br>24.2% in US<br>31.2% in Canada<br>31.1% in Mexico		Relatively stable:<br>26.2% in DM popularity (2009-2014) [2]<br>28.4% in DM popularity (1988-1994) [2]
IgAN	7.6 (Europe) [3]<br>45 (Japan) [4]	25.3 per million (Europe) [3]	21-22 per million in 2021 [5]	542-627 per million in 2021[5]
LN	4.5 (Denmark) [6]	64 per million (Denmark) [6]	10 per million between 1976 and 2018 (increasing trend from 7 to 13 over this period) [7]	Increasing trend from 168 per million in 1985 to 212 per million in 2015 [7]
FSGS			7 per million in general population [8]<br><br>19.6 per million in veterans (2000-2020) [9]	164.7 per million in veterans (2000-2020) [9]
MN	10-12 [10]	199 per million in UK [11]	10 per million in Minnesota (1994-2003) [12]	

DN, diabetic nephropathy; DM, diabetic mellitus; IgAN, IgA nephropathy; LN, lupus nephritis; FSGS, focal segmental glomerulosclerosis; MN, membranous glomerulonephritis.

2 Supplementary Figures

Figure S1. A visual representation of Google Trends searches used for glomerular diseases.

Figure S2. Monthly data on global search trends for five glomerular diseases.

Figure S3. Monthly data on search trends for five glomerular diseases in the United States.

Figure S4. Search Activity Trends for the Term IgAN in Global and the Top Five English-Speaking Countries with the Highest Search Popularity

Figure S5. Search Activity Trends for the Term FSGS in Global and the Top Five English-Speaking Countries with the Highest Search Popularity

Figure S6. Search Activity Trends for the Term DN in Global and the Top Five English-Speaking Countries with the Highest Search Popularity

Figure S7. Search Activity Trends for the Term LN in Global and the Top Five English-Speaking Countries with the Highest Search Popularity

Figure S8. Search Activity Trends for the Term MN in Global and the Top Five English-Speaking Countries with the Highest Search Popularity

Figure S9. Search interest in five glomerular diseases across various states in the United States.

2.1 Figure S1. A visual representation of Google Trends searches used for glomerular diseases.

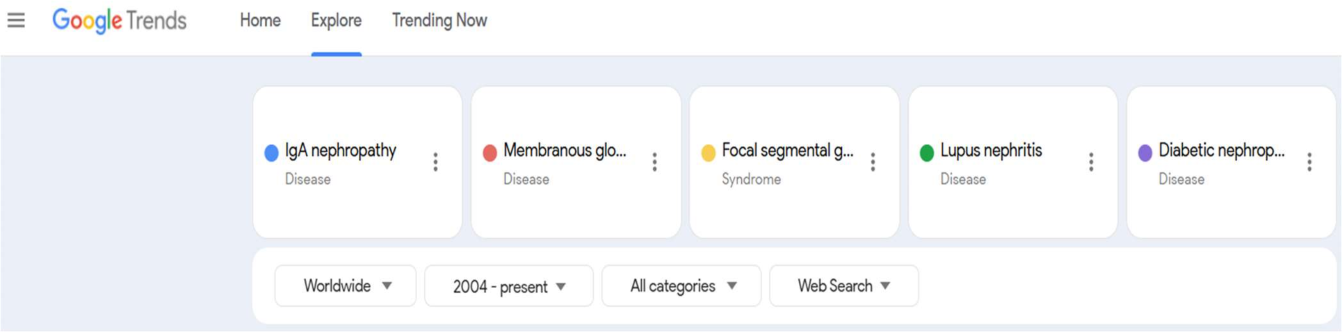

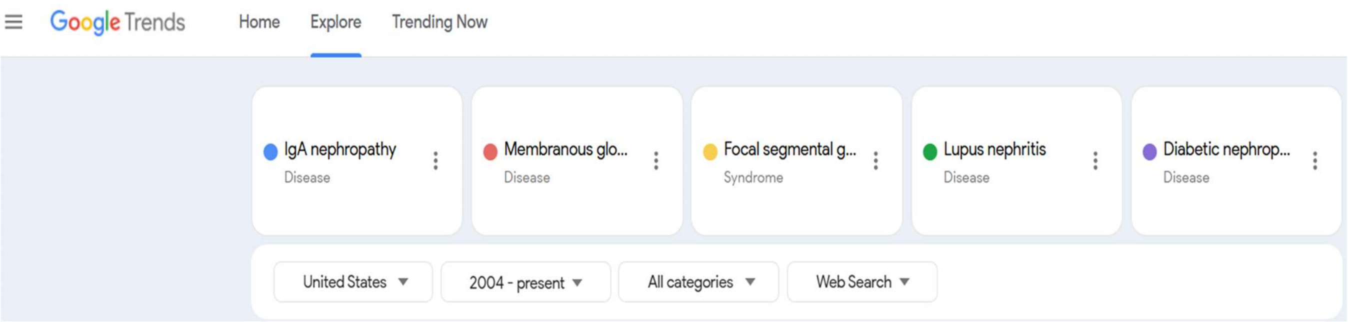

2.2 Figure S2. Monthly data on global search trends for five glomerular diseases.

A. Monthly data on global search trends for glomerular diseases disclosed by Google Trends. **1).** The Augmented Dickey–Fuller (ADF) stationarity test indicated that the search trends over the time series for FSGS and DN is stationary (ADF statistic = -6.305, p<0.0001 for FSGS; ADF statistic = -3.437, p=0.01 for DN), IgAN, MN and LN are not stationary (ADF statistic = -2.138, -0.2.238, and -1.439, p=0.229, 0.193, and 0.563, respectively). The comparative analysis on global monthly data confirms that there are statistically significant (p<0.01) differences in the trends across these diseases. The trends for FSGS and IgAN are declining, while the trends for LN and MN are increasing. DN has a relatively flat trend, indicating little to no change over time. **2).** Trends analysis for the next 12 months was performed using Time Series Forecasting. The forecasts and 95% confidence intervals are displayed. The predicted trends indicated no notable increases across the five glomerular diseases studied. **B.** Average monthly interest. One Way ANOVA test. p<0.001 across these diseases. IgAN: IgA nephropathy, MN: membranous glomerulonephritis, FSGS: focal segmental glomerulosclerosis, LN: lupus nephritis, DN: diabetic nephropathy.

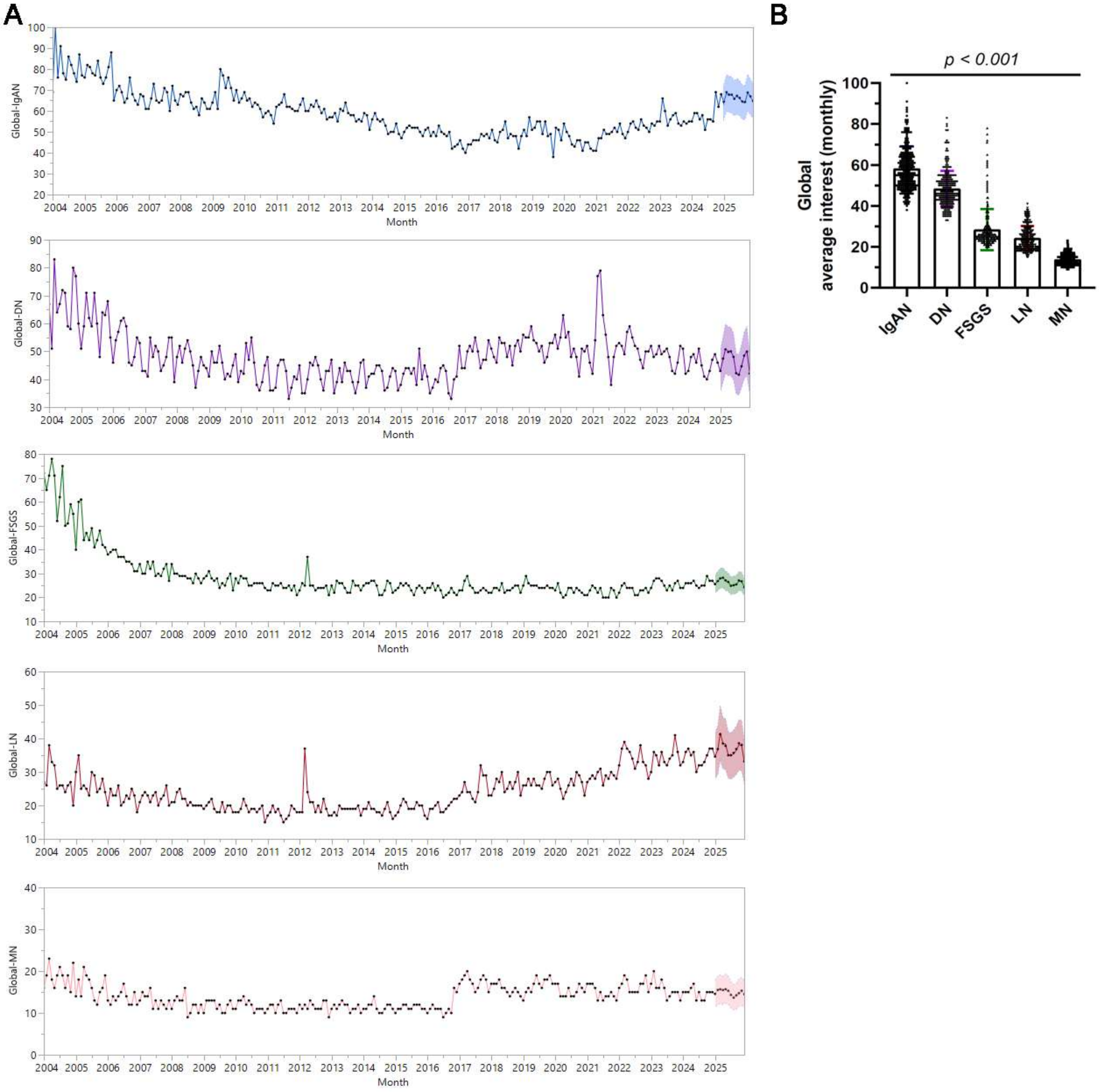

2.3 Figure S3. Monthly data on search trends for five glomerular diseases in the United States.

A. Monthly data on search trends for five glomerular diseases in the United States. **1).** The Augmented Dickey–Fuller (ADF) stationarity test indicated that the search trends over the time series for MN and DN are stationary (ADF statistic = -2.889 and -13.782, p=0.047 and <0.0001, respectively), LN and FSGS are potentially stationary at the 10% level (ADF statistic = -2.783 and -2.345, p=0.068 and 0.158, respectively). IgAN is not stationary (ADF statistic = -1.857, p=0.352). The comparative analysis on monthly data indicates statistically significant (*p<0.001*) differences in the trends of the five glomerular diseases in the United States. IgAN and FSGS show decreasing trends, while LN shows increasing trends. DN and MN have a relatively flat trend, indicating little to no change over time. **2).** Trends analysis for the next 12 months was performed using Time Series Forecasting. The forecasts and 95% confidence intervals are displayed. The predicted trends indicated no notable increases across the five glomerular diseases studied. **B.** Average monthly interest. One Way ANOVA test. *p<0.0001 vs IgAN, #p<0.0001 vs FSGS, Δp<0.0001 vs DN and LN. IgAN: IgA nephropathy, MN: membranous glomerulonephritis, FSGS: focal segmental glomerulosclerosis, LN: lupus nephritis, DN: diabetic nephropathy.

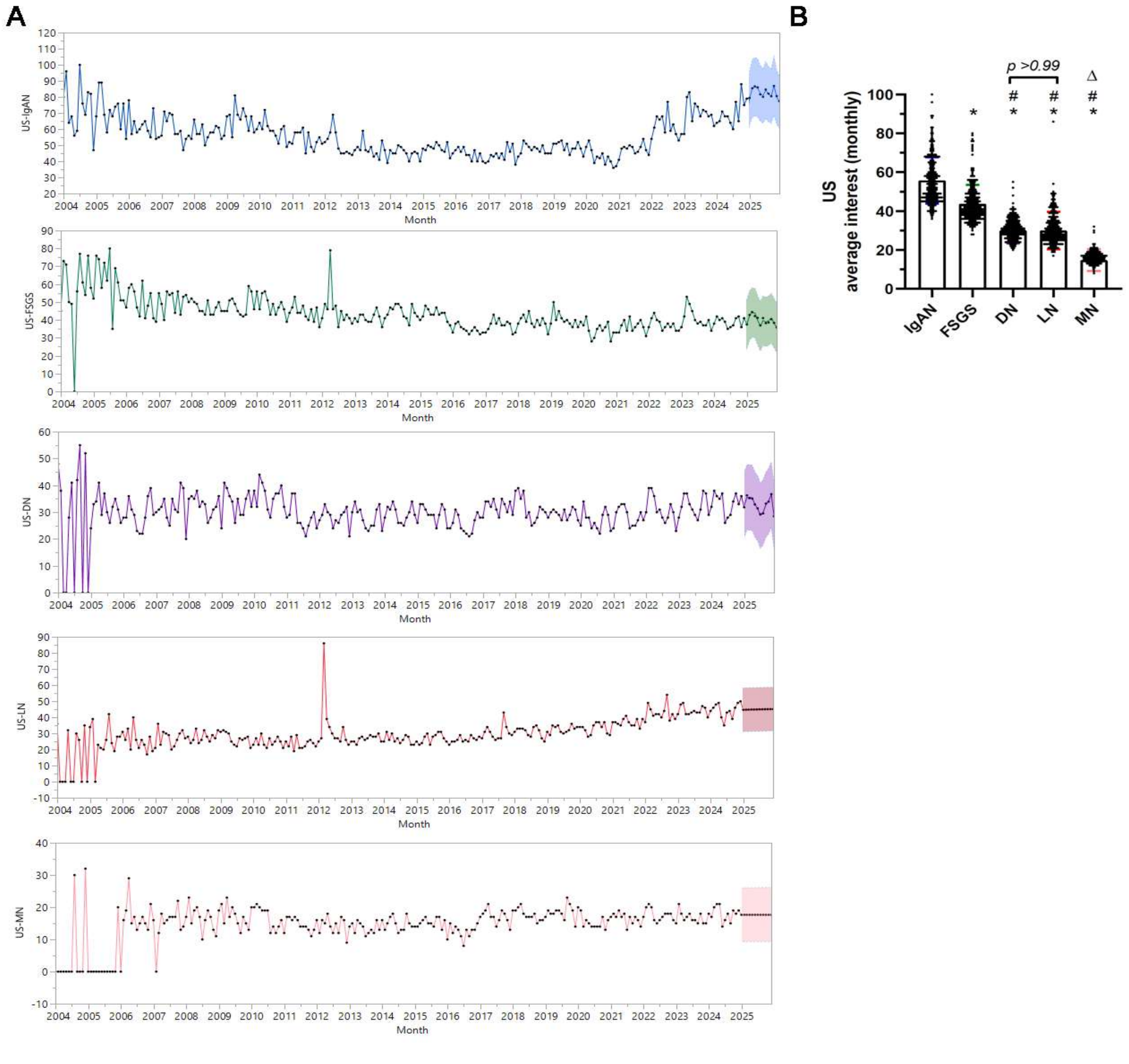

2.4 Figure S4. Search Activity Trends for the Term IgAN in Global and the Top Five English-Speaking Countries with the Highest Search Popularity

In the context of IgAN, the US, UK, Canada, Australia, and Ireland emerged as the top five English-speaking countries where the term was most frequently searched. The highest search activity was observed in the USA, UK, Canada, and Australia. In the US, search activity closely mirrored global trends. The Pearson correlation coefficient between the Global and US data is approximately 0.73, indicating a moderate positive correlation and suggesting similar trends across these datasets. Notable variations in search activity were evident before 2011 in the UK, Canada, and Australia. Although search interest in Ireland was generally lower, it experienced significant fluctuations during the specified period.

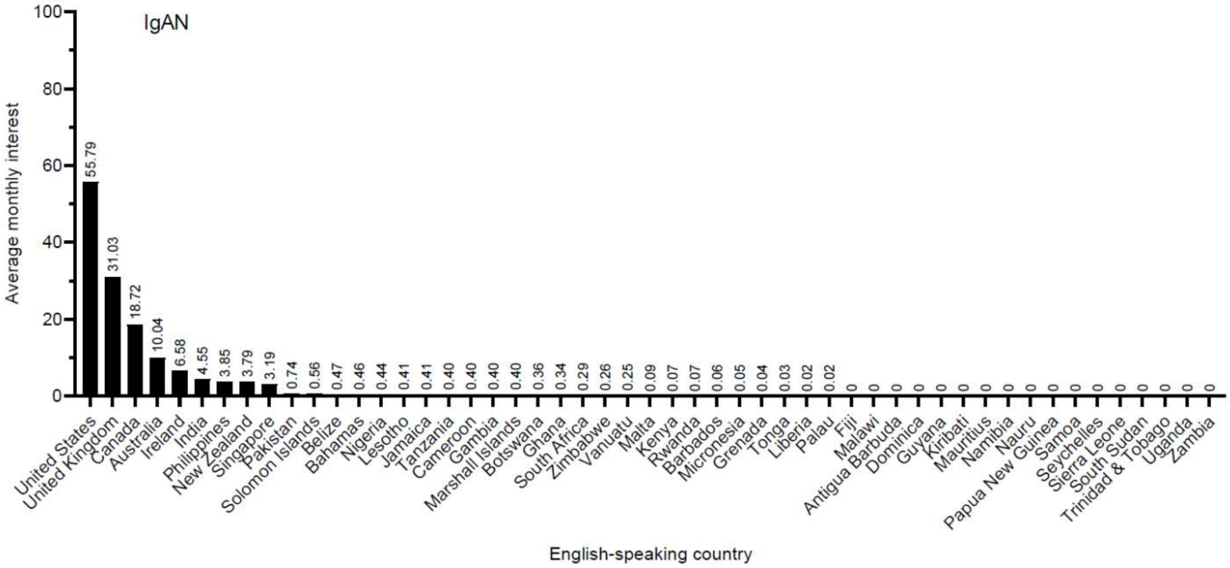

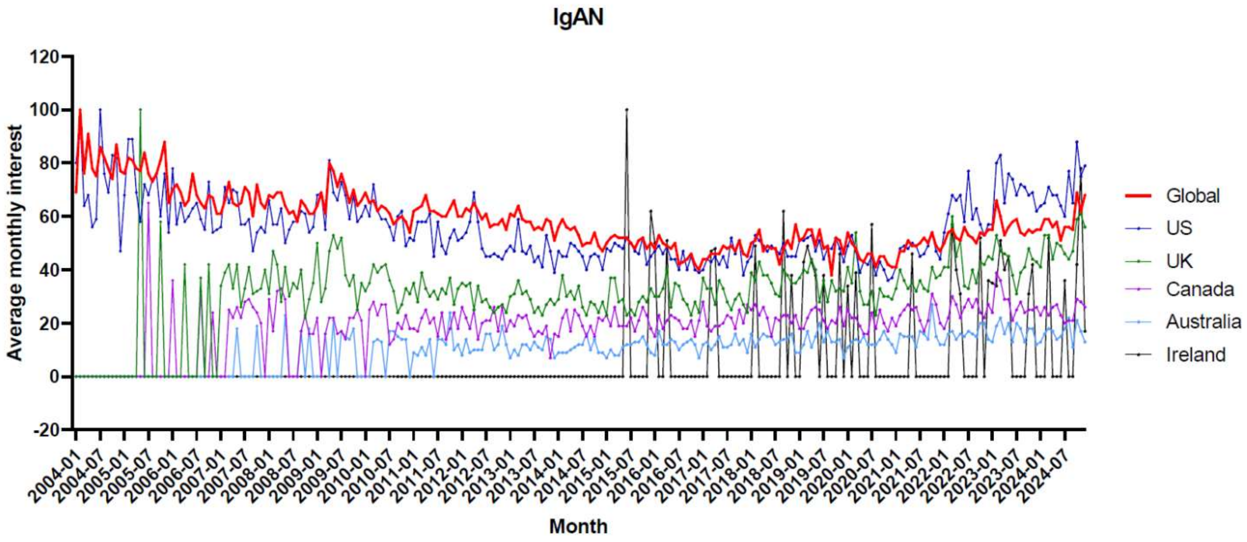

2.5 Figure S5. Search Activity Trends for the Term FSGS in Global and the Top Five English-Speaking Countries with the Highest Search Popularity

For FSGS, the primary English-speaking countries with the highest search popularity were the US, UK, Canada, Australia, and India. The bulk of the search activity centered in the USA, where the Relative Search Index (RSI) was higher than the global average. The Pearson correlation coefficient between the Global and US data is approximately 0.65, indicating a moderate positive correlation and pointing to similar patterns between these datasets. Search activity in the UK, Canada, Australia, and India were significantly lower, with notable fluctuations occurring before 2012. After this period, the RSI in these countries remained below 20.

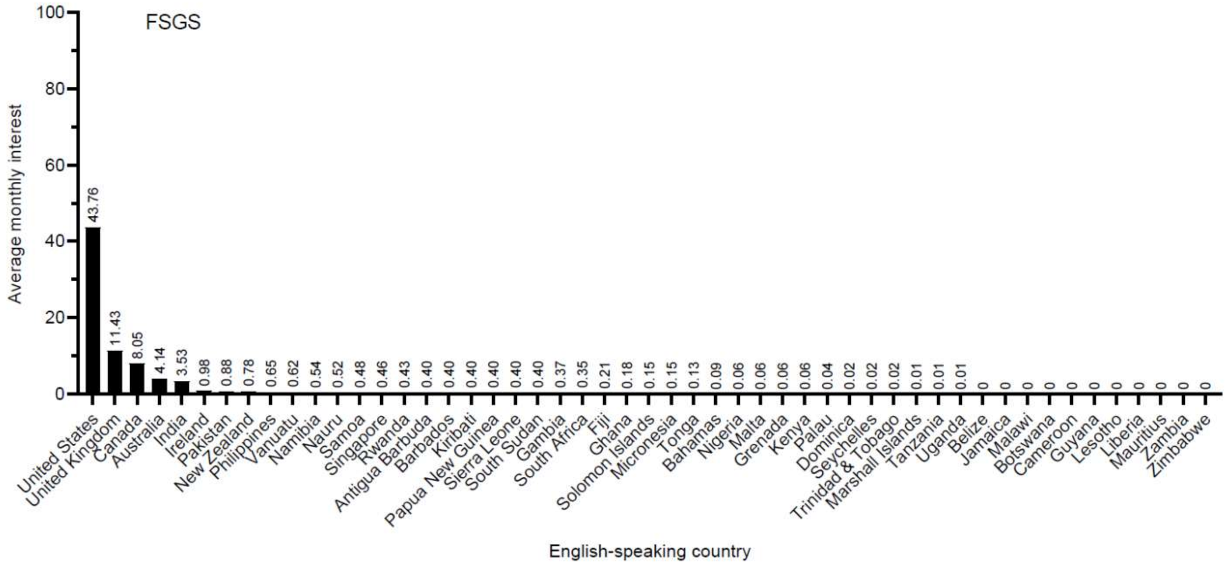

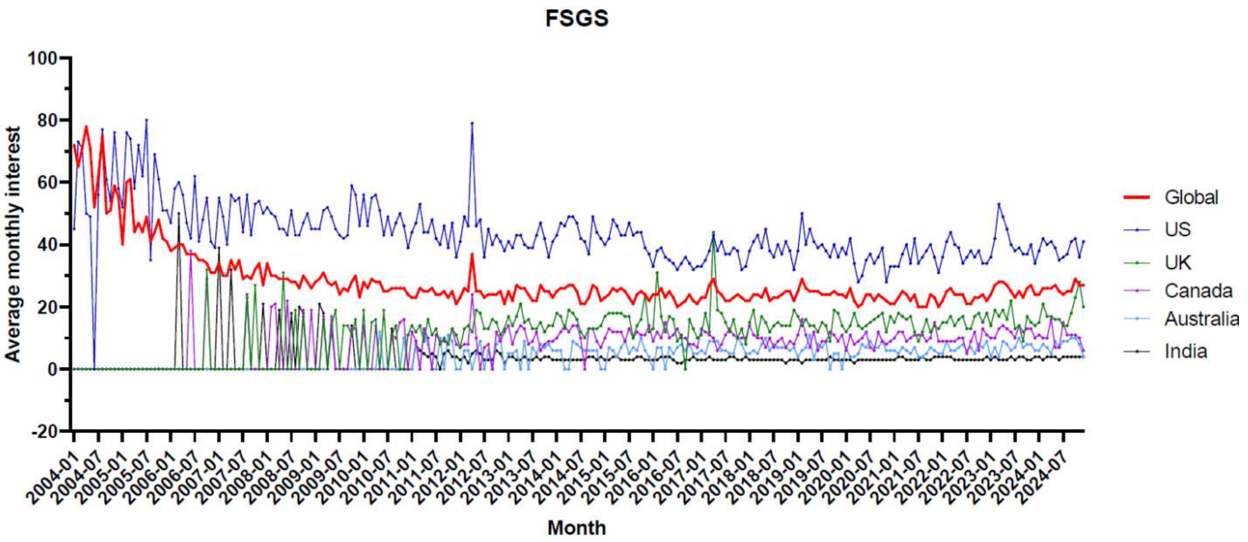

2.6 Figure S6. Search Activity Trends for the Term DN in Global and the Top Five English-Speaking Countries with the Highest Search Popularity

For DN, the US, UK, India, Canada, and Australia were identified as the top five English-speaking countries with the highest frequency of searches for the term. All search activity was lower than the global trends. The Pearson correlation coefficient between the Global and US data is approximately 0.40, suggesting a weak positive correlation within these datasets.

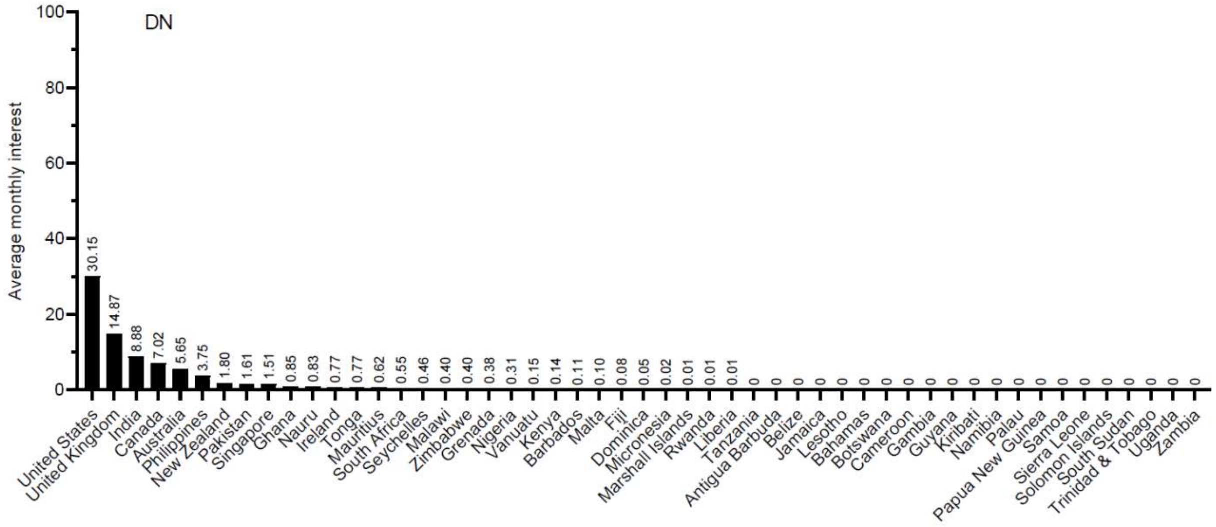

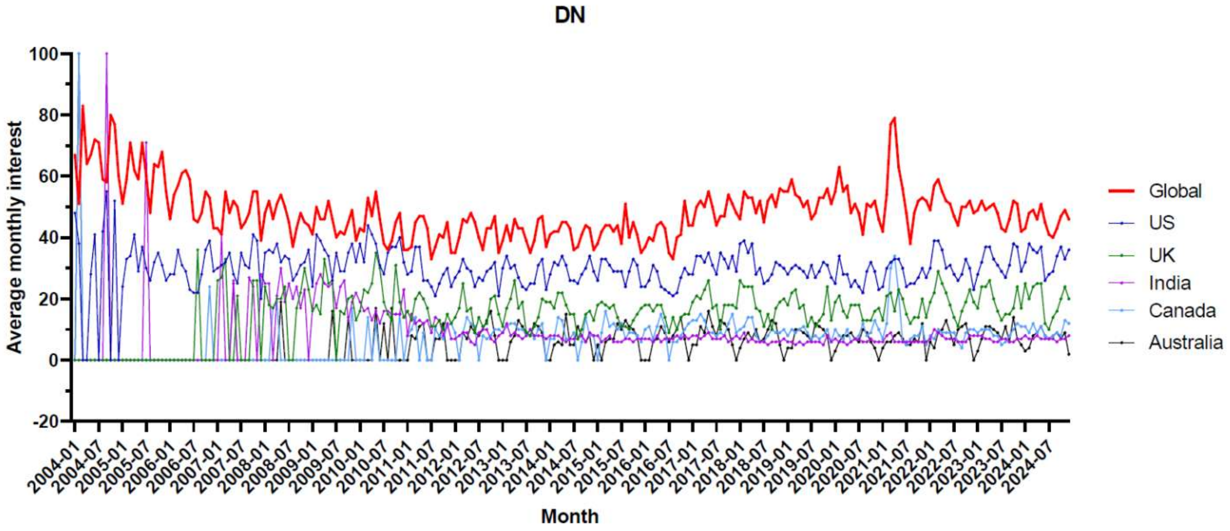

2.7 Figure S7. Search Activity Trends for the Term LN in Global and the Top Five English-Speaking Countries with the Highest Search Popularity

For LN, the US, UK, Canada, India, and Philippines were identified as the top five English-speaking countries with the highest frequency of searches for the term. Search activity was generally low across these countries. In the US, search trends closely aligned with global patterns. The Pearson correlation coefficient between the Global and US data is approximately 0.62, suggesting a moderate positive correlation and pointing to similar trends within these datasets. In other four countries, the Relative Search Index (RSI) was below 20.

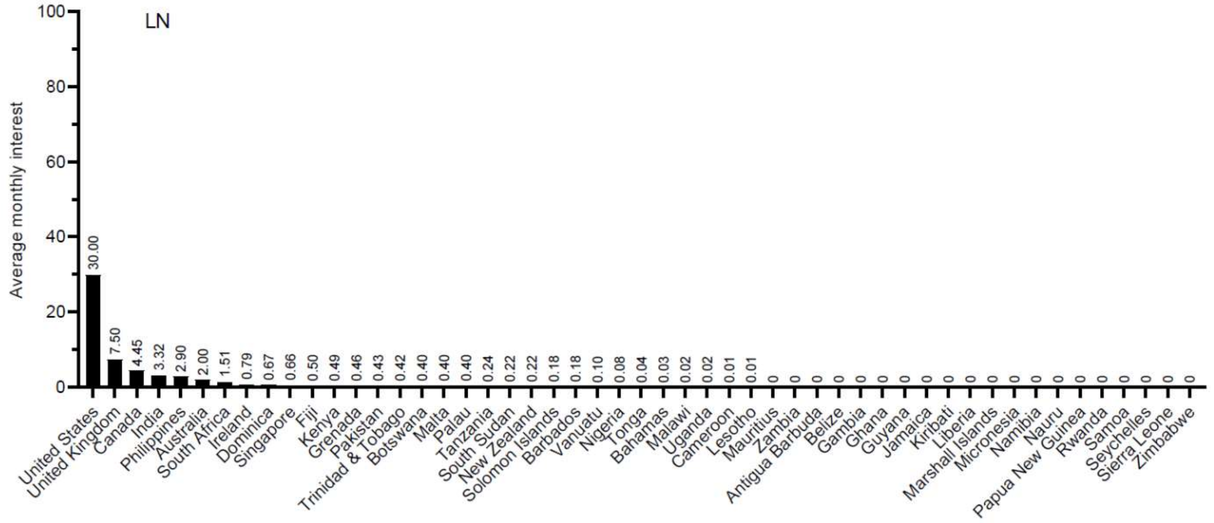

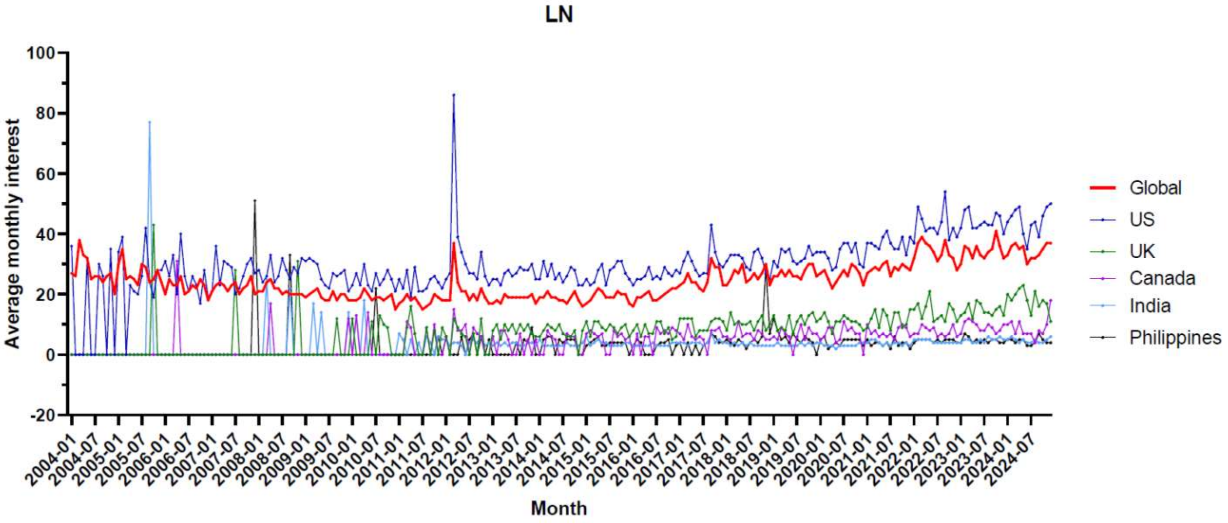

2.8 Figure S8. Search Activity Trends for the Term MN in Global and the Top Five English-Speaking Countries with the Highest Search Popularity

In the case of MN, the US, UK, Canada, India, and Australia were identified as the top five English-speaking countries with the highest frequency of searches for the term. Search activity was generally low across these countries, with the Relative Search Index (RSI) remaining below 30. In the US, search trends closely aligned with global patterns post-2007. The Pearson correlation coefficient between the Global and US data is approximately 0.58, suggesting a moderate positive correlation and pointing to similar trends within these datasets.

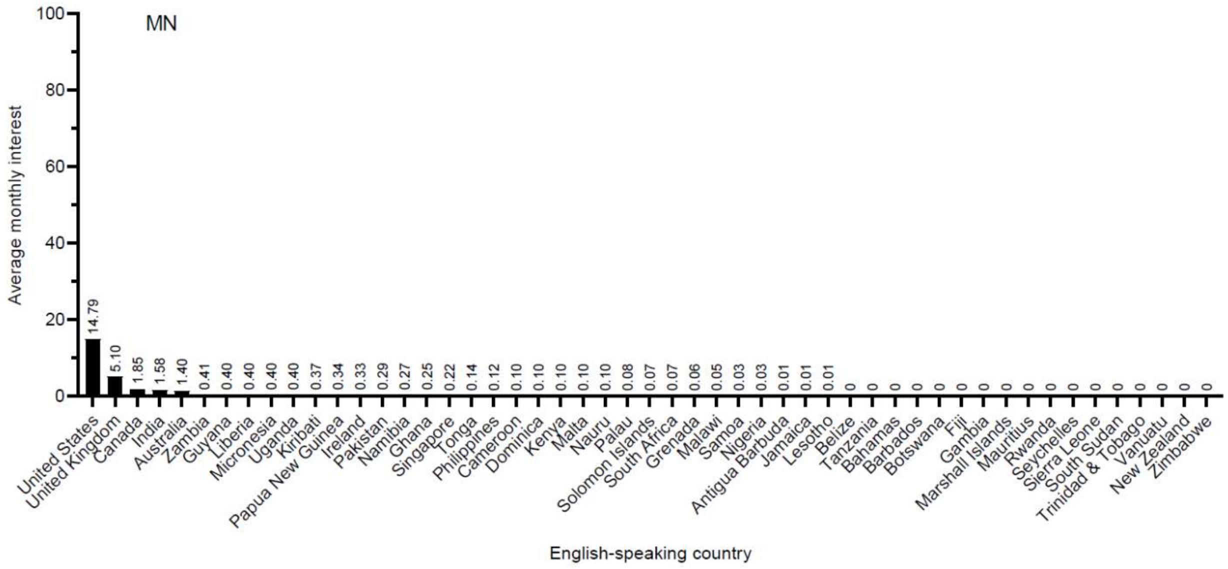

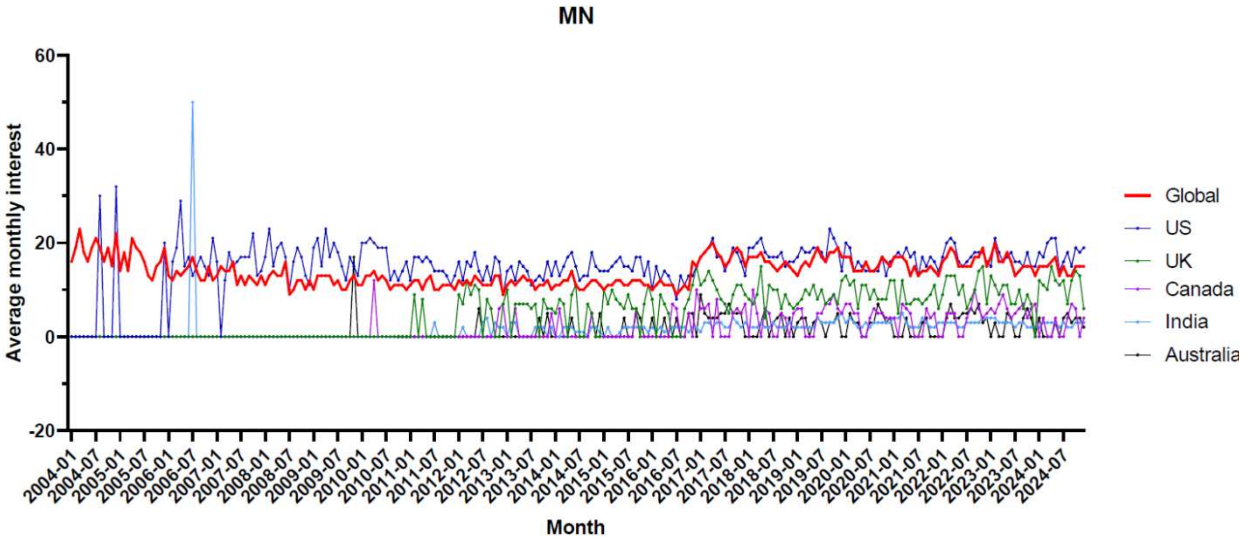

2.9 Figure S9. Search interest in five glomerular diseases across various states in the United States.

Interest by subregion: See in which location the term was most popular during the specified time frame. Values are calculated on a scale from 0 to 100, where 100 is the location with the most popularity as a fraction of total searches in that location, a value of 50 indicates a location which is half as popular. A value of 0 indicates a location where there was not enough data for this term. **Note:** A higher value means a higher proportion of all queries, not a higher absolute query count. So, a tiny country where 80% of the queries are for "bananas" will get twice the score of a giant country where only 40% of the queries are for "bananas".

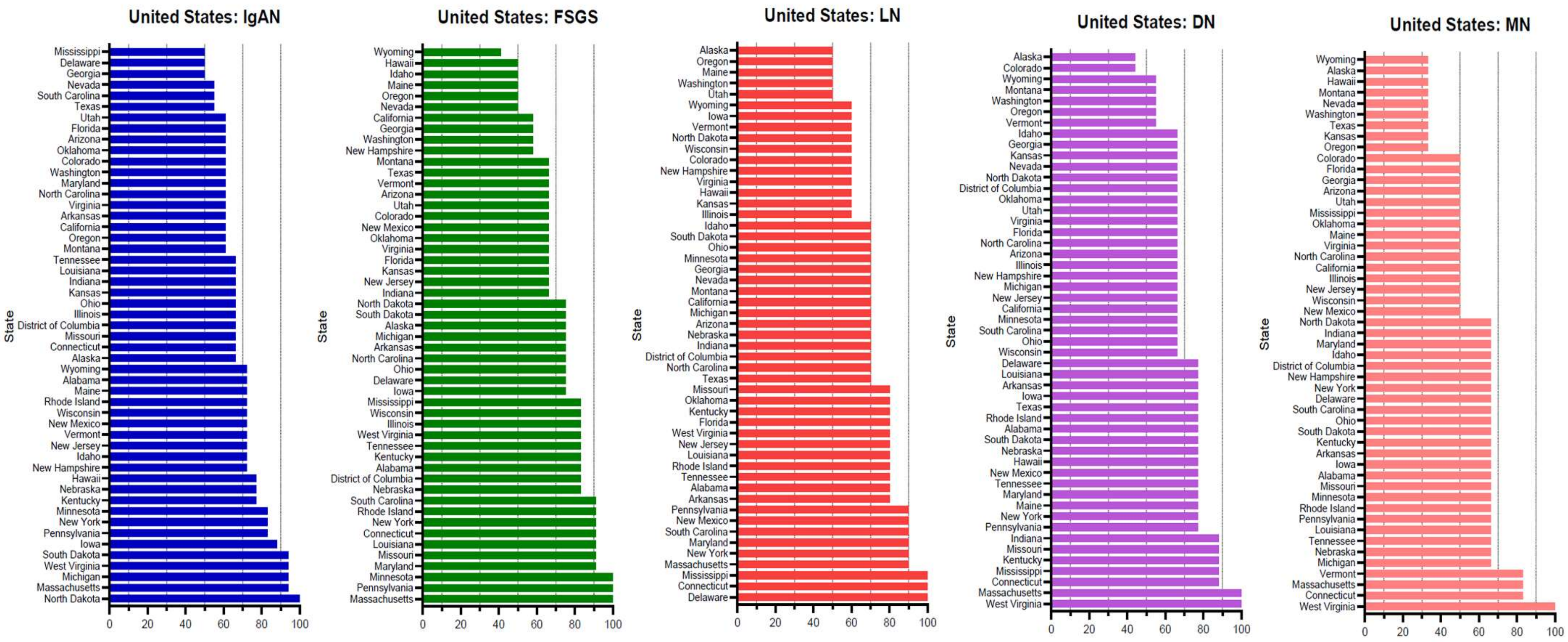

References

1 Zahra, S. *et al.* Prevalence of nephropathy among diabetic patients in North American region: A systematic review and meta-analysis. *Medicine (Baltimore)* **103**, e39759 (2024). https://doi.org:10.1097/MD.0000000000039759

2 Afkarian, M. *et al.* Clinical Manifestations of Kidney Disease Among US Adults With Diabetes, 1988-2014. *JAMA* **316**, 602-610 (2016). https://doi.org:10.1001/jama.2016.10924

3 Willey, C. J. *et al.* The incidence and prevalence of IgA nephropathy in Europe. *Nephrol Dial Transplant* **38**, 2340-2349 (2023). https://doi.org:10.1093/ndt/gfad082

4 Schena, F. P. & Nistor, I. Epidemiology of IgA Nephropathy: A Global Perspective. *Semin Nephrol* **38**, 435-442 (2018). https://doi.org:10.1016/j.semnephrol.2018.05.013

5 DeCongelio, M., Ali, S. N., Furegato, M., Bhattacharjee, S. & Fernandes, A. W. The incidence and prevalence of immunoglobulin A nephropathy in the United States. *Clin Nephrol* (2024). https://doi.org:10.5414/CN111489

6 Hermansen, M. L., Lindhardsen, J., Torp-Pedersen, C., Faurschou, M. & Jacobsen, S. Incidence of Systemic Lupus Erythematosus and Lupus Nephritis in Denmark: A Nationwide Cohort Study. *J Rheumatol* **43**, 1335-1339 (2016). https://doi.org:10.3899/jrheum.151221

7 Hocaoglu, M. *et al.* Incidence, Prevalence, and Mortality of Lupus Nephritis: A Population-Based Study Over Four Decades Using the Lupus Midwest Network. *Arthritis Rheumatol* **75**, 567-573 (2023). https://doi.org:10.1002/art.42375

8 Sprangers, B., Meijers, B. & Appel, G. FSGS: Diagnosis and Diagnostic Work-Up. *Biomed Res Int* **2016**, 4632768 (2016). https://doi.org:10.1155/2016/4632768

9 Goldschmidt, D. *et al.* Epidemiology and burden of focal segmental glomerulosclerosis among United States Veterans: An analysis of Veteran's Affairs data. *PLoS One* **19**, e0315302 (2024). https://doi.org:10.1371/journal.pone.0315302

10 McGrogan, A., Franssen, C. F. & de Vries, C. S. The incidence of primary glomerulonephritis worldwide: a systematic review of the literature. *Nephrol Dial Transplant* **26**, 414-430 (2011). https://doi.org:10.1093/ndt/gfq665

11 Hamilton, P. *et al.* Membranous nephropathy in the UK Biobank. *PLoS One* **18**, e0281795 (2023). https://doi.org:10.1371/journal.pone.0281795

12 Swaminathan, S. *et al.* Changing incidence of glomerular disease in Olmsted County, Minnesota: a 30-year renal biopsy study. *Clin J Am Soc Nephrol* **1**, 483-487 (2006). https://doi.org:10.2215/CJN.00710805
